# Effects on tumor growth and immunosuppression of a modified Tα1 peptide along with its circular dichroism spectroscopy data

**DOI:** 10.1016/j.dib.2018.07.058

**Published:** 2018-07-29

**Authors:** Fanwen Wang, Bin Li, Pengcheng Fu, Qingqing Li, Heng Zheng, Xingzhen Lao

**Affiliations:** a School of Life Science and Technology, China Pharmaceutical University, Nanjing 210009, PR China; b Department of Neurology, the First People׳s Hospital of Chenzhou, Hunan 423000, PR China

## Abstract

The data presented in this article are related to the research article entitled “Immunomodulatory and Enhanced Antitumor Activity of a Modified Thymosin α1 in Melanoma and Lung Cancer” (Wang et al., 2018). Tα1 has been evaluated as effective in cancer treatment. In order to make it capable to target tumor, a peptide iRGD was introduced to Tα1. The anti-tumor activity was accessed by constructing in vivo melanoma and human non-small-cell lung cancer models treated with Tα1-iRGD to measure the tumor volume over time and tumor weight at the last day. The concentration of IFN-γ and IL-2 in C57BL/6 mice peripheral blood was determined by ELISA. And the immunomodulatory ability of Tα1-iRGD was evaluated in vivo by thymus index and spleen index. Those functions this paper was aimed at may have relationship with its secondary structure, so the circular dichroism spectra of Tα1, iRGD and Tα1-iRGD was performed.

**Specifications Table**

**Table:** 

Subject area	Biology, Immunology
More specific subject area	Cancer, Immunity, Protein structure
Type of data	Figure
How data was acquired	Survey, the circular dichroism spectra of peptides were recorded at 25 °C on a Jasco J-815 CD Spectropolarimeter at 190–250 nm using 0.1 cm path-length quartz cuvette
Data format	Analyzed
Experimental factors	N/A
Experimental features	in vivo anti-tumor experiments, in vivo immune experiments, secondary structure determination
Data source location	Nanjing, China
Data accessibility	The data are supplied with this article

**Value of the data**

• The effectiveness of Tα1-iRGD in melanoma and lung cancer model was evaluated for the first time.
• Tα1-iRGD exerted a stronger immunomodulatory activity compared to Tα1.
• Retention of helix structure in Tα1 has a relationship with the biological activity of fusion protein.

## 1 Data

Data shown in B16F10 and H460 tumor models provide information about the change of tumor volume and body weight of C57BL/6 mice or BALB/c nude mice over time, also, the tumor weight of different groups on the last day. Tα1, as an immunomodulator used in cancer therapy [1], has abilities to induce the activation of DCs and T cells as well as the secretion of interferon-gamma (IFN-γ) and interleukin-2 (IL-2) [2]. IFN-γ is important in the immune system stems for its immunomodulatory activity, whereas IL-2 can promote the differentiation of T cells into effector T cells and boost the host immunity against cancer [3], [4]. Thus, the standard curve data of IFN-γ and IL-2 in melanoma model, and the absorbance of samples treated with Tα1-iRGD were determined and can be found in different sheets.

Tα1 can antagonize the decrease of thymus index and spleen index in immunosuppression models induced by hydrocortisone (HC) [5]. The constructed immunosuppressant models were used to determine the effects of iRGD introduction on immunomodulatory activity. Body weight of ICR mice, the spleen weight and thymus weight were measured after executed.

Circular dichroism (CD) spectroscopy is usually used to investigate the secondary structure of proteins in solution because of their dextrorotary and levorotary components. In this article, mean residue ellipticity of Tα1, iRGD and Tα1-iRGD in different wavelength was showed.

## 2 Experimental design, materials and methods

### 2.1 The tumor-bearing model

#### 2.1.1 Experimental design

Construct tumor model and monitor the tumor growth after treatment of different peptides to evaluate the antitumor activity.

#### 2.1.2 Materials

The mouse melanoma cell line B16F10 and the human lung cancer cell line H460 were purchased from the American Type Cell Culture (Shanghai, China). Paclitaxol (Taxol) was provided by Jiangsu Yew Pharmaceutical Company Limited (Wuxi, Jiangsu Province, China). C57BL/6 mice and Balb/c nude mice were purchased from the Comparative Medicine Center of Yangzhou University (China). Mouse IFN-γ enzyme-linked immunosorbent assay (ELISA) kit and mouse IL-2 ELISA kit were purchased from Shanghai Bioye Biological Technology Co., Ltd. The anti-CD8, anti-CD86 and anti-CD31 antibodies were purchased from Abcam. All experimental procedures involving animals were performed strictly in accordance with the Interdisciplinary Principles and Guidelines for the Use of Animals in Research and the National Institutes of Health Guide for the Care and Use of Laboratory Animals (NIH Publications No. 8023, revised 1978) and were approved by the Jiangsu Provincial Experimental Animal Management Committee under Contract 2016(su)-0010.

#### 2.1.3 Methods

**Fig. 1:**
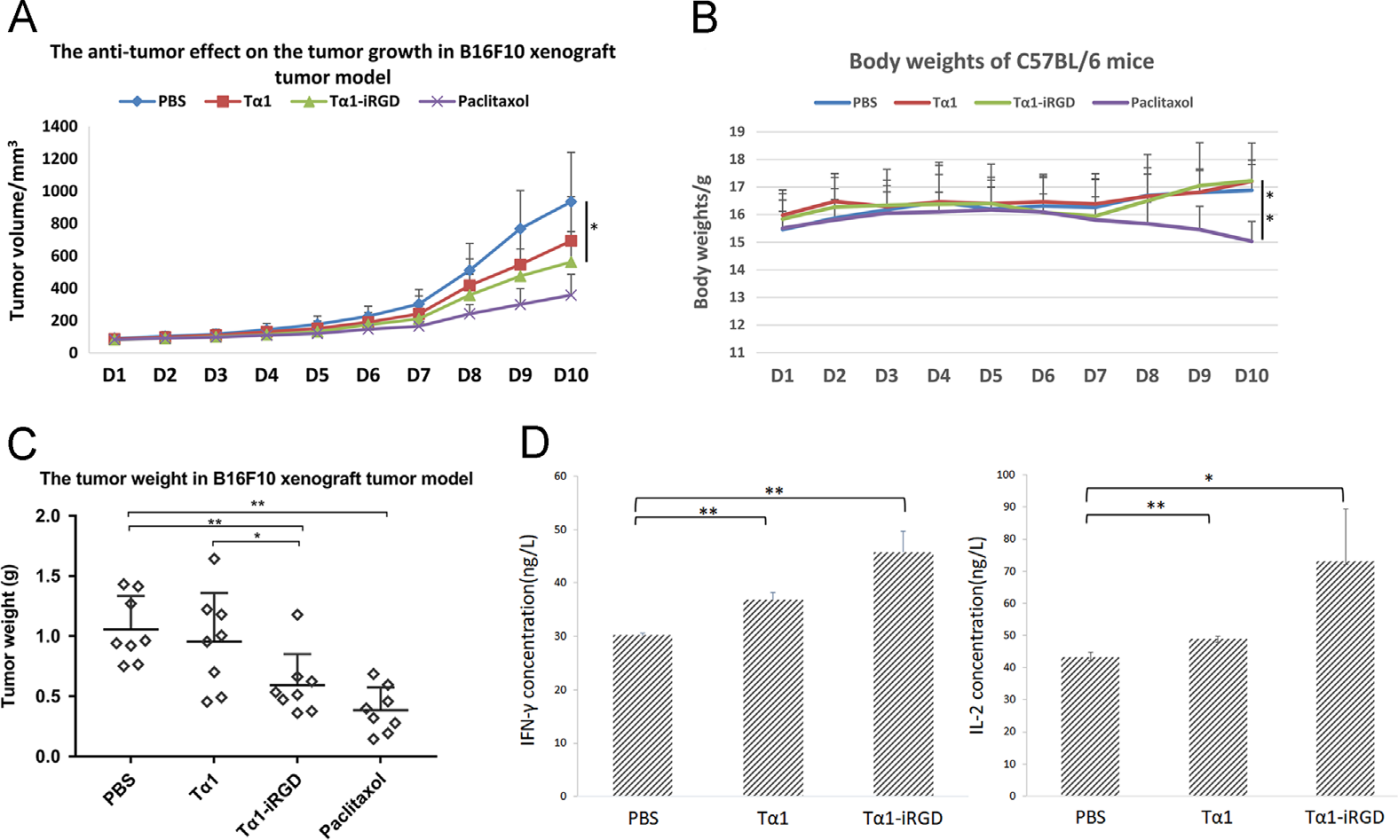
Tumor treatment with Tα1-iRGD in C57BL/6 mice (*n* = 8). The mice bearing the B16F10 melanoma model were subcutaneously injected with Tα1-iRGD or Tα1 at a dose of 0.0815 µmol /kg. PBS and paclitaxol were used as negative and positive controls, respectively. (A). The tumor volume. (B). The body weights of C57BL/6. (C). The tumor weights. (D). The concentration of IFN-γ and IL-2 in peripheral blood. Date were analyzed using one-way ANOVA followed by post hoc Tukey HSD test using R Software Version 3.3.1.; Error bars, mean ± SEM; n.s., not significant; *p< 0.05; **p< 0.01; ***p< 0.001.

**Fig. 2:**
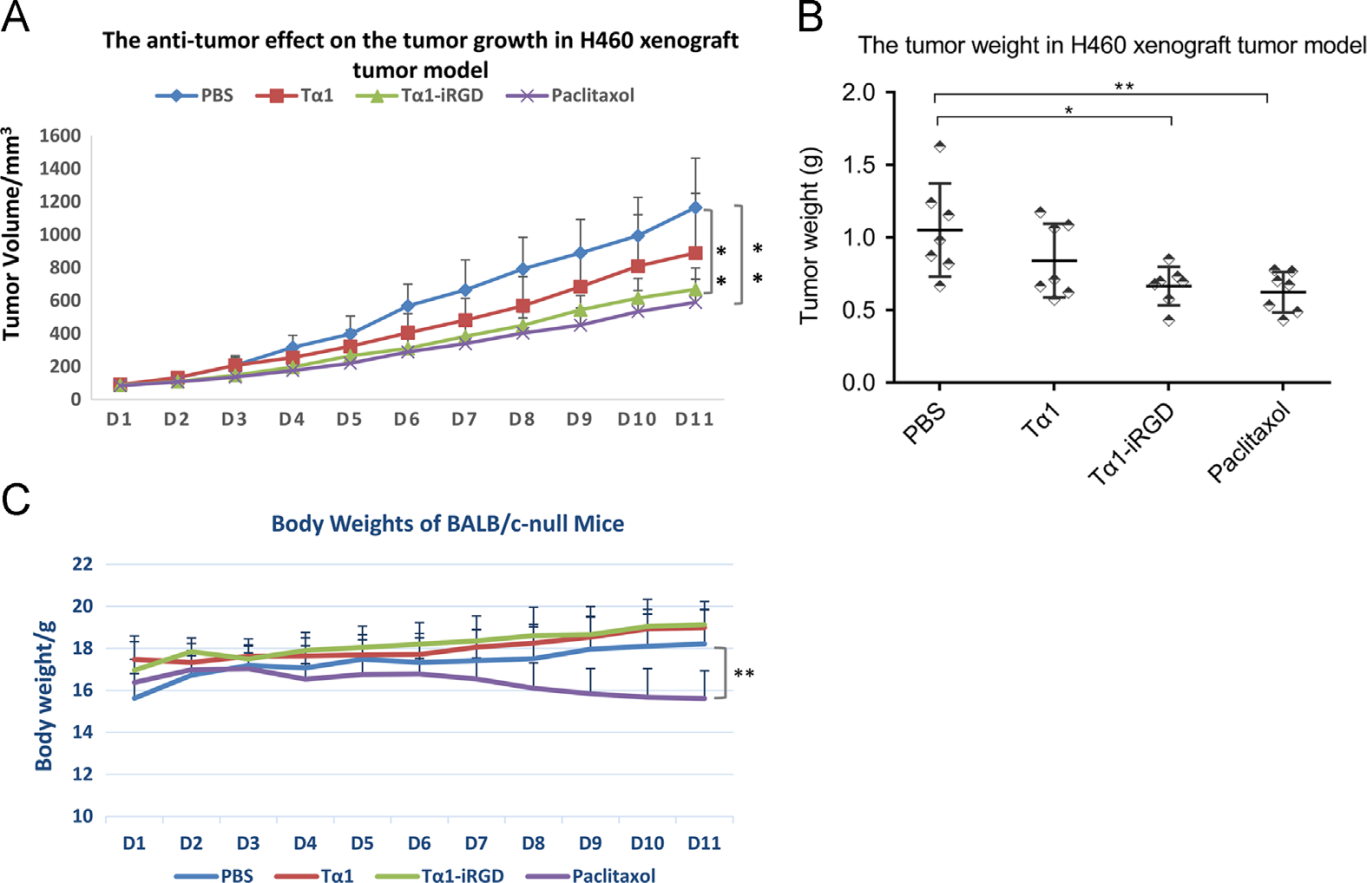
Suppression of H460 human lung cancer by Tα1-iRGD or Tα1 in BALB/c nude mice (*n* = 7). Mice bearing H460 human lung tumor were subcutaneously injected with 0.25 mg/kg (the dose of 0.0815 µmol of Tα1 is equivalent to 0.25 mg) of Tα1 or Tα1-iRGD at the dose of 0.25 mg Tα1-equiv./kg (0.34 mg/kg) in 0.1 mL of PBS once daily for 10 days. PBS and paclitaxol were used as negative and positive controls, respectively. (A). The tumor volume. (B). The tumor weights. (C). The body weights of BALB/c. Date were analyzed using one-way ANOVA followed by post hoc Tukey HSD test using R Software Version 3.3.1.; Error bars, mean ± SEM; n.s., not significant; *p< 0.05; **p< 0.01; ***p< 0.001.

B16F10 cells and H460 cells were incubated in RPMI 1640 medium with 10% fetal bovine serum and 1% Penicillin-Streptomycin at 37 °C, 5% CO_2_. To generate the tumor model, the C57BL/6 mice (5–6 weeks old) were injected B16F10 cancer cells (~5 × 10^5^ cells/mouse) and the female BALB/c nude mice (5–6 weeks old) were injected H460 cancer cells (1 × 10^7^ cells/mouse) into the mid-left or right side. When the tumor size reached 80 mm^3^, mice were randomly separated into four different groups: the negative control group (PBS, everyday), the positive control group (10 mg/kg Tax, once every two days), Tα1 group (0.25 mg/kg, everyday) and Tα1-iRGD (0.34 mg/kg, everyday). Tα1 and Tα1-iRGD were dissolved in PBS and filtered by 0.22 µm membrane. The solution volume each treatment was 0.1 mL s.c. The mice were euthanized when the average tumor volume of PBS group reached 1000 mm^3^, and in melanoma models, peripheral blood was taken to stand for at least 30 min, centrifuged at 4000 rpm for 10 min, and then determined by using a mouse IFN-γ ELISA kit and a mouse IL-2 ELISA kit. Their tumors were weighed and taken for further biomarker analysis, i.e., histochemistry (H&E) staining and immunohistochemical (IHC) staining for CD8 and CD86 in B16F10 model or CD31 in H460 model. All data are analyzed and showed in Fig. 1 (melanoma models) and Fig. 2 (H460 cancer models).

### 2.2 Immunosuppressant model

#### 2.2.1 Experimental design

Construct Immunosuppressant model, measure the mice body weight after treatment of different peptides and assess the recovery of immune organs (thymus and spleen) in mice.

#### 2.2.2 Materials

The ICR (SPF) mice were purchased from the Comparative Medicine Center of Yangzhou University (China). Hydrocortisone (HC) was purchased from Anshan Fengyuan Pharmaceutical Co., Ltd. (Anhui, China).

#### 2.2.3 Methods

**Fig. 3:**
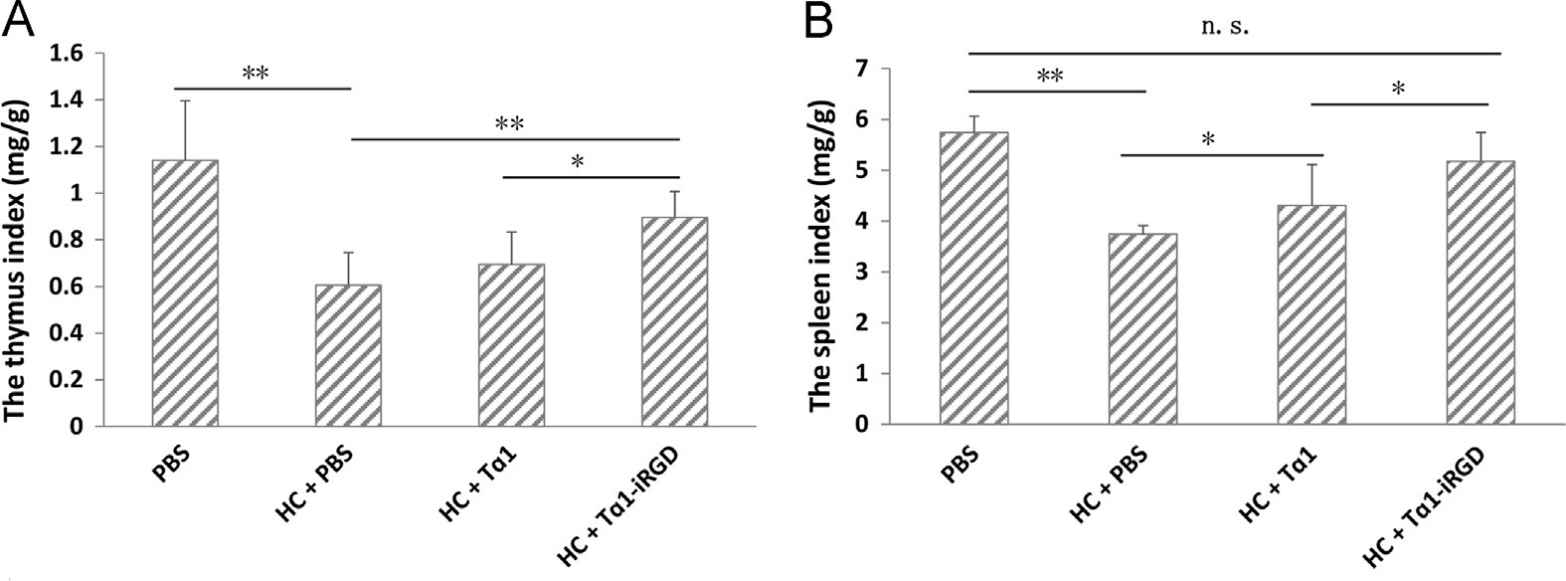
Thymus and spleen indices of immunosuppressed mice treated with PBS, Tα1, or Tα1-iRGD for 7 days (*n* = 7). The immunomodulatory activity of Tα1-iRGD and Tα1 in vivo was evaluated by a hydrocortisone-induced immunosuppression model. The dose of Tα1-iRGD and Tα1 was 0.0815 µmol/kg in this experiment. (A). The thymus indices. (B). The spleen indices. Date were analyzed using one-way ANOVA followed by post hoc Tukey HSD test using R Software Version 3.3.1.; Error bars, mean±SEM; n.s., not significant; *p< 0.05; **p< 0.01; ***p< 0.001.

The ICR mice were randomly divided into four groups (7 mice/group). The normal control group (PBS group) received 0.1 mL PBS via subcutaneous (s.c.) injections once daily for 14 consecutive days. The model and two other treatment groups were first rendered immunosuppressant by hydrocortisone (HC, 50 mg/kg of body weight s.c.) once daily for 7 consecutive days to construction of immunosuppressant mice. Thereafter, the model group ("HC+PBS" group) were injected s.c. injection of 0.1 mL PBS once daily for 7 consecutive days, and the two other treatment groups ("HC+Tα1" group and "HC+Tα1-iRGD " group) were injected s.c. with 0.0815 µmol/kg of Tα1 or Tα1-iRGD in 0.1 mL PBS once daily for 7 consecutive days. On the last day, the mice were weighed and their blood samples were drawn for further analysis. The mice were finally euthanized and the spleen and thymus were collected and then weighed to calculate the spleen and thymus indexes after treatment (Fig. 3).

### 2.3 Circular dichroism spectroscopy

#### 2.3.1 Experimental Design

Dissolve peptide in different solution, perform circular dichroism spectra to assess the impact of iRGD on Tα1 structure.

#### 2.3.2 Methods

**Fig. 4:**
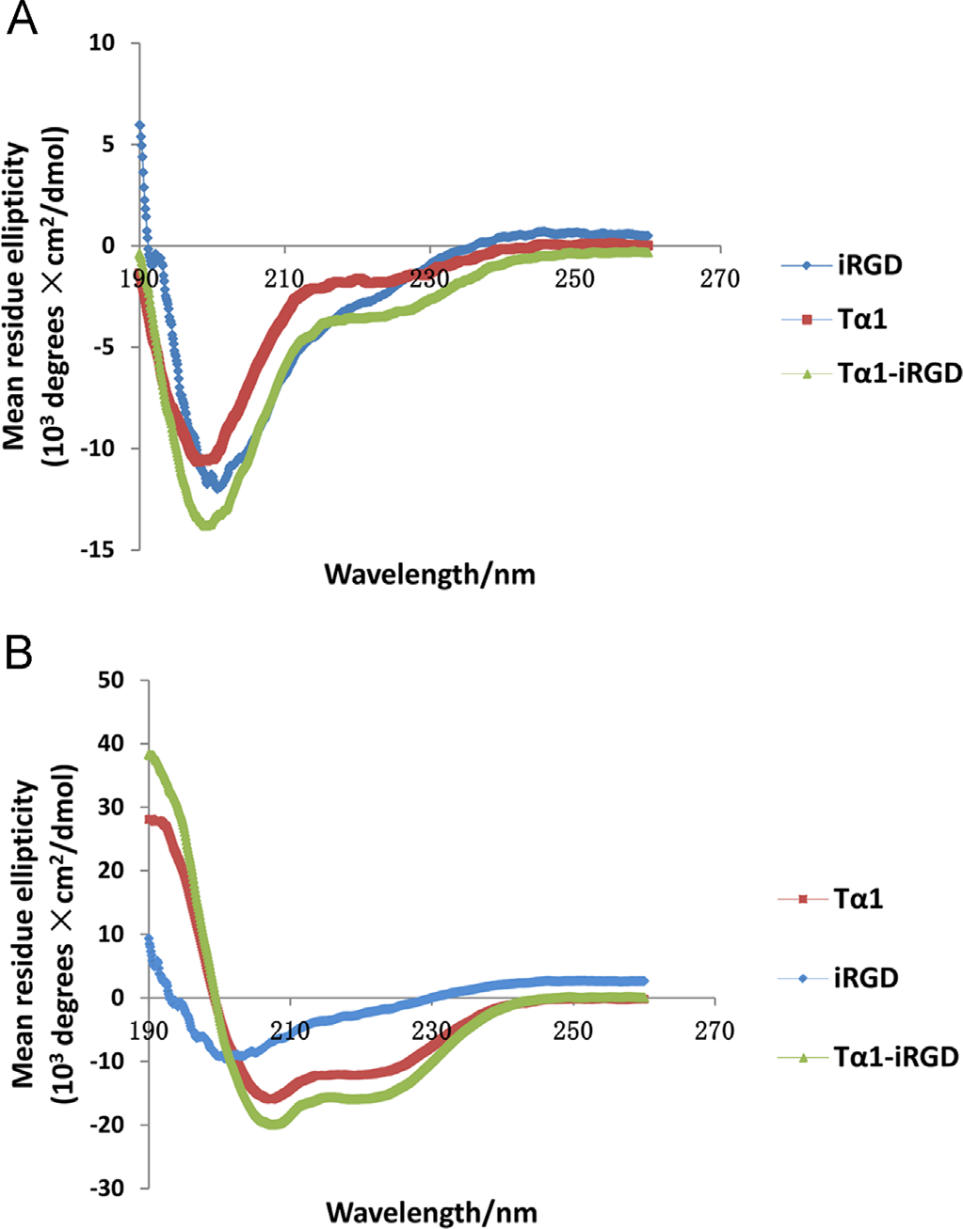
Circular dichroism spectra of Tα1-iRGD, Tα1, and iRGD. (A). CD spectra of peptides in water; all peptides showed an unstructured, random coil-like conformation. (B). CD spectra of peptides in water/TFE (50:50). Tα1-iRGD and Tα1 both showed an induced helical structure in the presence of TFE. iRGD remained to exhibit the unstructured, random coil-like conformation.

Tα1, Tα1-iRGD, or iRGD were prepared in pure water or 50% water/ 50% 2,2,2 trifluoroethanol (TFE) mixture to a final concentration of 0.25 mg/mL. The circular dichroism spectra of the three peptides were recorded at 25 °C on a Jasco J-815 CD Spectropolarimeter at 190–250 nm using 0.1 cm path-length quartz cuvette. The scans were conducted at 100 nm/min. The spectra of the buffer for peptide samples were subtracted for blank control. Finally, the CD data were analyzed using Jasco software and shown as Fig. 4.

## References

[1] Danielli R. (2012). Thymosin alpha1 in melanoma: from the clinical trial setting to the daily practice and beyond. Ann. N. Y. Acad. Sci.

[2] Wolf G.T. (1989). Interleukin 2 receptor expression in patients with head and neck squamous carcinoma. Effects of thymosin alpha 1 in vitro. Arch. Otolaryngol. Head Neck Surg..

[3] Setrerrahmane S., Xu H. (2017). Tumor-related interleukins: old validated targets for new anti-cancer drug development. Mol. Cancer.

[4] Sennikov S.V. (2017). Modern strategies and capabilities for activation of the immune response against tumor cells. Tumour Biol..

[5] Goldstein A.L., Badamchian M. (2004). Thymosins: chemistry and biological properties in health and disease. Expert Opin. Biol. Ther..

