# Supplementary material for: Effects on tumor growth and immunosuppression of a modified Tα1 peptide along with its circular dichroism spectroscopy data
Source: Data Brief. 2018 Jul 29;20:126–31. doi: 10.1016/j.dib.2018.07.058 (PMC6088563; doi:10.1016/j.dib.2018.07.058)
Supplement: Supplementary file 1 — Supplementary material. [file mmc1.docx]

**Conflict of interest**

There is no conflict of any financial or non-financial interest.
